# Using organic fertilizers to increase crop yield, economic growth, and soil quality in a temperate farmland

**DOI:** 10.7717/peerj.9668

**Published:** 2020-08-19

**Authors:** Yu Cen, Liyue Guo, Meizhen Liu, Xian Gu, Caihong Li, Gaoming Jiang

**Affiliations:** 1 State Key Laboratory of Vegetation and Environment Change, Institute of Botany, Chinese Academy of Sciences, Beijing, China; 2 University of Chinese Academy of Sciences, Beijing, China

**Keywords:** Rapeseed meal, Soybean meal, Cattle manure, Crop yield, Soil chemical characteristics, Soil aggregate, Microbial biomass, PLFA, Soil quality

## Abstract

We used a constant total N application base rate to conduct a two-year field experiment comparing the effects of three organic fertilizers (rapeseed meal (RSM), soybean meal (SBM), and cattle manure (CM)) on the crop yield, economic growth, and soil quality of a winter wheat-summer maize rotation system. Winter wheat and summer maize in rapeseed meal treatment (RSMT), soybean meal treatment (SBMT), and cattle manure treatment (CMT) showed yield increases of 161%, 299%, and 256%, respectively, when compared to no organic fertilizer treatment (CK) (*P* < 0.05). The annual net incomes of SBMT and CMT were 1.46 and 1.42 times higher, respectively, than RSMT. Compared to the results of the CK group, RSM, SBM, and CM stimulated the soil physically, chemically, and biologically. We found the highest soil macroaggregate proportions, soil organic matter (SOM) levels, total N (TN) levels, and phospholipid fatty acid (PLFA) levels in SBMT. The highest soil pH, microbial biomass carbon (MBC) levels, and microbial biomass nitrogen (MBN) levels were observed in CMT. We used a soil quality index (SQI) to evaluate soil quality. After the two-year fertilization treatments, we calculated the SQI using a minimum data set (MDS). We used SOM levels and actinomycete quantity for the MDS properties. The SQI values were significantly different across the four treatments, with the highest values occurring in SBMT, then CMT and RSMT. In conclusion, SBM and CM were more effective than RSM at maintaining crop yield, economic growth, and soil quality.

## Introduction

The world population has reached 7.6 billion people and is expected to reach 8.6 billion by 2030, 9.8 billion by 2050, and 11.2 billion by 2100 (Gerliani, Hammami & Aïder, 2019; Tripathi et al., 2019). To meet the global anticipated food demand caused by this population growth, various agricultural practices have been proposed and tested (Godfray et al., 2010), including the excessive application of chemical fertilizers, pesticides, and herbicides (Ju et al., 2007); large-scale utilization of water resources (Tilman et al., 2002); and intensive plowing (Koch & Stockfisch, 2006). Although these practices can increase food production, they also pose serious problems, including the decrease in organic matter content (Koch & Stockfisch, 2006; García-Orenes et al., 2012; Fernández-Romero et al., 2016), soil acidification (Guo et al., 2010; Zhu et al., 2018), greenhouse gas emissions (McGill, 2015), and loss of biodiversity (Clark & Tilman, 2008; Yu et al., 2015). Many reports have emphasized the need for major changes to the agricultural system to meet the challenge of feeding the growing population while minimizing environmental impacts (Godfray et al., 2010; Foley et al., 2011). Organic farming, which largely relies on organic materials instead of chemical fertilizers, pesticides, herbicides, or other synthetics (Luttikholt, 2007), has been increasingly recognized by both researchers and consumers (Ma & Joachim, 2006; Chen et al., 2014). High-efficiency organic fertilizers can increase crop yield without depleting soil quality, making their application a means of supporting both long-term food security and environmental preservation.

Previous studies have shown that the periodic application of organic amendments in an organic farming system can increase soil macroaggregate proportions (Zhang et al., 2020), soil organic matter (SOM) and total N (TN) levels (Liu et al., 2016; Guo et al., 2016), earthworm density (Guo et al., 2016; Meng et al., 2016), microbial biomass carbon (MBC) levels, microbial biomass nitrogen (MBN) levels, and enzymatic activities (Liu et al., 2010), which help to maintain crop yield and food quality (Liu et al., 2016; Tejada & Benítez, 2020). Although organic farming has numerous advantages, many farmers are still hesitant to change their systems, primarily due to the concern that their yields may be reduced during the conversion from conventional farming (Reganold et al., 2001; Tu et al., 2006). Additionally, using organic materials such as crop residues, compost, and poultry manure requires a great amount of human labor. Therefore, it is essential for organic farming development to identify new and better organic fertilizers that both minimize crop yield losses and enhance soil quality during the conversion period. Traditional organic fertilizers mainly include human excreta (Akram et al., 2019), compost, and poultry manure (Guo et al., 2016; Liu et al., 2016). Ecologists have suggested more available, effective, and cheaper organic fertilizers to completely or partially replace chemical fertilizers, such as different types of straw (Yang et al., 2015; Zhao et al., 2018), biochar (Zhang et al., 2020), sewage sludge, and municipal solid waste (Tejada & Benítez, 2020). Soybean meal (SBM) and rapeseed meal (RSM) are the residues created after oil is extracted from soybean (*Glycine max* (Linn.) Merr.) and rapeseed (*Brassica campestris* Linn.) seeds. They are high in N, which helps to increase vegetable, fruit, tea, and tobacco crop yields (Peng et al., 2008; Shan et al., 2010; Liu et al., 2012; Barker et al., 2019). However, few studies have investigated the effects of using SBM and RSM to fertilize a winter wheat (*Triticum aestivum* L.)-summer maize (*Zea mays* L.) rotating plantation.

According to data collected from the National Bureau of Statistics of China, the annual average sown areas of wheat and maize over the past 5 years were 24.5 and 43.3 million ha, respectively. Additionally, the annual yields of wheat and maize were 5,386.2 and 5,976.7 kg ha^−1^, respectively. The North China Plain (NCP) is a major grain production region containing about 35 million ha of croplands, 40% of which are dominated by a winter wheat-summer maize rotation system. This accounts for 61% and 45% of China’s total wheat and maize production, respectively (Yang et al., 2015). It is crucial to study the ecological and economic effects of SBM and RSM application in winter wheat-summer maize rotation systems in the NCP.

Soil quality is an indicator of sustainable soil management (Herrick, 2000), defined as “the capacity of soil to function within ecosystem boundaries to sustain productivity, maintain environmental quality, and promote plant and animal health” (Doran & Parkin, 1994). Fertilizers and soil management practices can affect soil quality by altering the physical, chemical, and microbiological indicators (Boafo et al., 2020). The most common method used to evaluate soil quality is the development of a soil quality index (SQI) (Qi et al., 2009; Cheng et al., 2016), which typically integrates the composite scores of multiple physical, chemical, and biological indicators (Andrews et al., 2002; Qi et al., 2009). The SQI method is considered to be effective at evaluating the effects of management practices on soil quality in agricultural lands (Zhang et al., 2004; Rojas et al., 2016; Nabiollahi et al., 2017; Boafo et al., 2020).

Fertilizer efficacy is typically evaluated by analyzing changes in crop yield and soil quality. In this study, we hypothesized that organic fertilizers could improve soil quality, crop yield, and the farmers’ profit. We carried out a two-year field experiment using a constant total N application rate (66.7 kg N ha^−1^ yr^−1^) from October 2017 to October 2019. Our objectives were to evaluate the effects of RSM, SBM, and cattle manure (CM) on a winter wheat-summer maize rotation system’s: (1) crop yield and economic growth; (2) physical, chemical, and biological soil characteristics; and (3) soil quality, evaluated using the SQI method.

## Materials and Methods

### Study area

**Figure 1:**
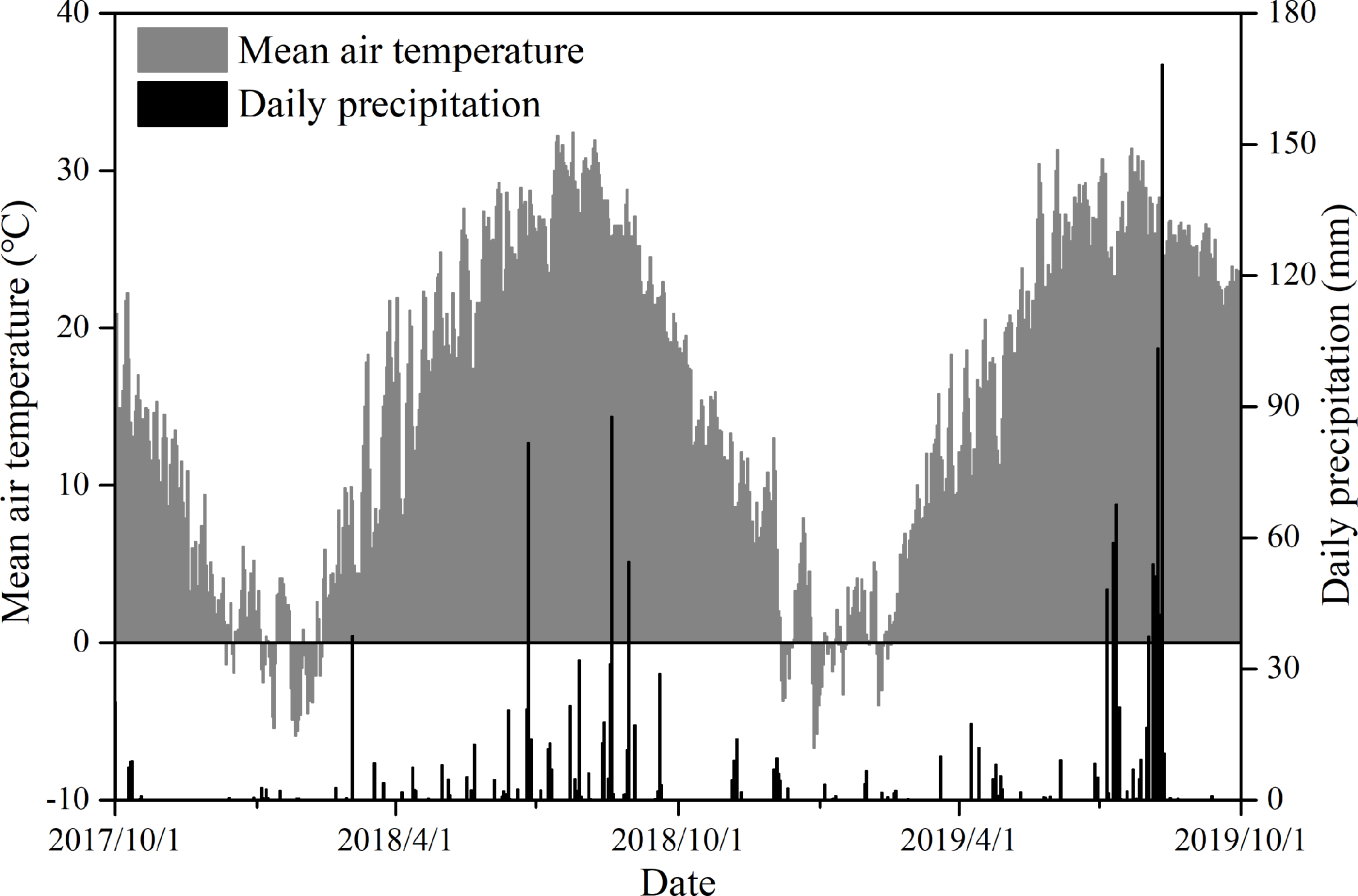
Daily precipitation (mm) and mean air temperature (°C) during the experimental period in the study area.

We conducted this experiment over two years in Jiang Jiazhuang Village, Pingyi County, Shandong Province, Eastern China (35°26′21″N, 117°50′11″E). Our study was approved by the Plant Eco-physiological Research Group (PERG) in the Institute of Botany, Chinese Academy of Sciences (project number: 201707.8). In this study, the name of the farmer is Jianjin Jiang who has a verbal agreement with the PERG in the Institute of Botany, Chinese Academy of Sciences. Although the farmland belongs to Jianjin Jiang, he has already rented it to the PERG. So, we should only get permission of the PERG. Any other permission from the individual and company is not needed. Shandong Province is an important source of food production in the NCP, and its main cropping system is a winter wheat-summer maize rotation. The study area has a typical temperate and monsoonal climate. In 2018 and 2019, the total annual rainfall was 685.1 mm and 842.2 mm, and the average mean air temperature was 15.2 °C and 15.1 °C, respectively. Figure 1 shows the daily precipitation and mean air temperature during our study from 1 October 2017 to 1 October 2019. The experimental soil was identified as an alfisol according to its soil taxonomy (IUSS Working Group WRB, 2014). The main soil (0–20 cm) characteristics are shown in Table 1.

**Table 1:** Properties of the experimental soil (0–20 cm) and the three organic fertilizers.

□	Water content (%)	OM (g kg^−1^)	TN (g kg^−1^)	TP (g kg^−1^)	TK (g kg^−1^)	pH
Soil	17.70 ± 0.47	15.41 ± 0.37	0.97 ± 0.01	0.81 ± 0.06	17.38 ± 0.54	6.95 ± 0.03
RSM	8.48 ± 0.38	791.49 ± 0.51	60.14 ± 0.06	20.64 ± 1.01	19.04 ± 0.67	□–
SBM	16.32 ± 0.54	798.60 ± 0.50	70.89 ± 0.12	11.21 ± 0.65	21.30 ± 0.78	□–
CM	65.02 ± 3.20	420.47 ± 20.11	18.67 ± 0.23	5.23 ± 0.32	17.78 ± 0.82	□–

**Notes.**

Data were means ± standard error (*n* = 3). RSM, SBM and CM represent rapeseed meal, soybean meal and cattle manure, respectively.

OM organic matter TN total nitrogen TP total phosphorus TK total potassium

### Experimental design

We set up our study using a randomized block design with four treatments and three replicates based on previous related research (Zhen et al., 2014; Guo et al., 2016). Each plot had an area of 80 m^2^ (10 m ×8 m). The four treatments were: (1) RSMT, rapeseed meal treatment, 1,212 kg ha^−1^ yr^−1^ (fresh weight) of RSM; (2) SBMT, soybean meal treatment, 1,125 kg ha^−1^ yr^−1^ (fresh weight) of SBM; (3) CMT, cattle manure treatment, 10,215 kg ha^−1^ yr^−1^(fresh weight) of CM; and (4) CK, no organic fertilizer treatment. We applied the fertilizers according to the local farmers’ average SBM application rate. We distributed equivalent amounts of N (66.7 kg ha^−1^ yr^−1^) across the three organic fertilizer treatments. The three organic fertilizers were solid, and their nutrient and water levels are shown in Table 1. All fertilizers were applied one time before winter wheat seeding. The winter wheat cultivar Shannong 32 was sown in mid-October and harvested in early June the following year. The summer maize cultivar Jinhai 5 was sown in mid-June and harvested in early October. 172.5 kg ha^−1^ and 37.5 kg ha^−1^ of wheat and maize seeds were planted, respectively. The distance between rows of wheat was 0.3 m, and the distances between rows and between individual maize were 0.6 m and 0.3 m, respectively. All other field management practices remained unchanged. A rotary tiller ridged the plots one time before winter wheat seeding at a plowing layer depth of 30 cm. Crops were irrigated twice during the winter wheat season. No irrigation was necessary during the summer maize season because of the abundance of rainfall. Crops were physically weeded twice during the winter wheat season and once during the summer maize season.

### Yield assessment

Three 1 m^2^ crops were randomly harvested from each plot to determine winter wheat yield on 7 June 2018 and 10 June 2019. Three replicates of 10 consecutive summer maize plants in the same row were harvested on 9 October 2018 and 8 October 2019. The seeds of the harvested winter wheat and summer maize had a water content of < 14%.

### Farm profitability

We recorded in detail each of the total inputs and outputs of the four treatments. The treated wheat and maize were sold online for $0.89/kg^−1^ and $0.59/kg^−1^, respectively. We used the annual average net income to assess economic benefits.

### Soil sampling

We sampled the soils for chemical and biological characteristic analysis on 9 October 2018 and 8 October 2019. We used a 5 cm soil auger to select five random sampling points in the 0–20 cm layer, and then mixed the samples together into one. All soils were immediately transported to the laboratory, where all visible stones, roots, and undegraded fertilizer were removed. We divided the soil samples into two groups. One group was air-dried for chemical analysis, and the other group was passed through a 10-mesh sieve for biological analysis.

### Soil chemical analysis

We passed a portion of the air-dried soil through a 10-mesh sieve to analyze its soil pH, available N (AN), available P (AP), and available K (AK). The other group of air-dried soils was passed through a 100-mesh sieve to analyze its SOM, TN, total phosphorus (TP), and total potassium (TK). We used a digital pH meter (PB-10, Sartorius, Germany) and a 1:2.5 soil:water ratio to measure soil pH. AN was determined using NaOH hydrolysis (Bao, 2000). We extracted AP by applying 0.5 M NaHCO_3_ (1:10, w/v) for 30 min, and determined colorimetrically using the molybdate method (Watanabe & Olsen, 1965). AK was measured using flame photometry (Schollenberger & Simon, 1945). We measured SOM using the dichromate oxidation method (Nelson & Sommers, 1996). TN was determined using the Kjeldahl method (Bremner & Mulvaney, 1982) and the FOSS Kjeltec 8200 (Foss, Hilleroed, Denmark). We used nitric-perchloric digestion (1:1) to extract TP and TK, and used EPA Method 3050 (USEPA, 1986) for analysis.

### Soil aggregate distribution

We gathered our soil samples for aggregate distribution analysis from five subsamples (20 cm ×20 cm ×20 cm) of each plot on 8 October 2019. In the laboratory, we gently broke apart the moist soil samples along their natural break points and removed all visible stones, roots, and undegraded fertilizer. The soil samples were then air-dried for aggregate distribution analysis.

The soil aggregates were separated using the wet-sieve method (Elliott, 1986), and four aggregate-size classes were determined (large macroaggregate, >2 mm; small macroaggregate, 0.25–2 mm; microaggregate, 0.053–0.25 mm; and silt + clay, <0.053 mm). A subsample of 100 g air-dried soil was placed on top of a 2 mm sieve gently slaked with deionized water for 5 min. We then passed the slaked soil sample through three sieves with different mesh sizes (2 mm, 0.25 mm, and 0.053 mm) by automatically moving the sieves up and down for 15 min. All fractions left on each sieve and the bottom pan were thoroughly rinsed and transferred to aluminum boxes. We dried and weighed all aggregates to determine their size class.

### Soil biological analysis

We analyzed MBC and MBN levels using a chloroform fumigation-direct extraction procedure. We estimated the levels using the difference between the fumigated and non-fumigated soil samples, and a conversion factor of 0.45 (Vance, Brookes & Jenkinson, 1987; Brookes, 1995). We extracted 25 g of fresh soil from each soil sample using 50 ml of 0.5 mol L^−1^ K_2_SO_4_ solution. The other 25 g of fresh soil was fumigated with chloroform at room temperature for 24 h, after being extracted with the K_2_SO_4_ solution. We immediately stored all extracts at −20 °C to be measured using a total organic carbon (TOC) analyzer (multi N/C 3100, Analytik Jena, Germany). We weighed out 10 g of fresh soil to measure its water content and to calculate its MBC and MBN levels.

Phospholipid fatty acid (PLFA) analysis was performed following the procedure described by Bossio et al. (1998). We extracted lipids from freeze dried soil samples at −80 °C using a single-phase chloroform-methanol-citrate buffer mixture (1:2:0.8, v/v/v). The PLFAs were then purified and identified using an Agilent 6850 gas chromatograph with the Sherlock Microbial Identification System (MIDI) V4.5 with the internal standard set at PLFA 19:0. In this study, the PLFA group was made up of three components (Bossio et al., 1998; Fierer, Schimel & Holden, 2003): (1) bacteria, most commonly 14:0, 15:0, 16:0, 17:0, and 18:0; gram-negative bacteria (G–) cy17:0, cy19:0, 15:1 ω6c, 16:1 ω7c, 16:1 ω9c, 17:1 ω8c, 18:1 ω7c, and 18:1 ω9c; and gram-positive bacteria (G+) i14:0, i15:0, a15:0, i16:0, a16:0, i17:0, and a17:0; (2) fungi, most commonly 18:2 ω6c, 18:3 ω6c, 20:1 ω9c (Joergensen & Wichern, 2008), and arbuscular mycorrhizae fungi (AMF) 16:1 ω5c (Olsson, 1999); and (3) actinomycetes 10Me16:0, 10Me17:0, and 10Me18:0.

### Soil quality evaluation

We assessed soil quality using the SQI method. The SQI was developed according to the following three steps: (1) selecting a minimum data set (MDS) for soil indicators, (2) scoring the MDS indicators, and (3) integrating the scored indicators into one SQI value (Qiu et al., 2019; Boafo et al., 2020).

We performed one-way analysis of variance on 18 soil indicators. All indicators showed significant differences (*P* < 0.05) across the four treatments and were included in the total data set (TDS). The TDS soil indicators were employed in principal component analysis (PCA) and selected the components with eigenvalues >1 as principal components (PCs). For each PC, we considered highly loaded indicators as those with absolute loading values within 10% of the highest factor loading. When a PC contained only one highly loaded indicator, this indicator was selected for the MDS. When a PC contained multiple indicators, we determined each indicator’s relevance using Pearson’s correlation analysis. If they were not correlated (*r* < 0.70), all highly loaded indicators were selected for the MDS. Otherwise, only the indicator with the highest sum of correlation coefficients (absolute value) was selected for the MDS (Qiu et al., 2019; Boafo et al., 2020).

After MDS selection, we used a linear scoring method to transform each soil indicator into a unitless score ranging from 0.00 to 1.00 (Boafo et al., 2020). Indicators were divided into two types according to their soil parameter concentration thresholds: “more is better” and “less is better”. In this study, considering the contributions of the MDS indicators, we only applied the “more is better” function:

(1) \begin{eqnarray*}Y= \frac{X}{{X}_{max}} \end{eqnarray*}

where *Y* stands for the indicator linear score, *X* is the soil indicator value, and *X*_*max*_ is the maximum value for each soil indicator.

We weighed the MDS indicators using the PCA results where the percentage of PC variation was divided by the total percentage of variation. We integrated the transformed indicator scores into the SQI using the following weighted equation:

(2) \begin{eqnarray*}SQI={\mathop{\sum \nolimits }\nolimits }_{i=1}^{\mathrm{n}}{W}_{i}{S}_{i}\end{eqnarray*}

where *SQI* is the soil quality index, *S*_*i*_ is the indicator score, *W*_*i*_ is the weight of each indicator, and n is the number of soil indicators in the MDS. In this study, higher SQI values reflected better organic fertilizer treatment outcomes.

### Statistical analyses

We performed statistical analysis using Microsoft Excel (2016) and SPSS 22.0 (IBM Corp, Armonk, New York, USA). Crop yield and soil property differences across the four treatments over the same year were determined using one-way analysis of variance and Duncan’s test. We used an independent sample *t*-test to assess the differences between different years, 2018 and 2019, over the same treatment. We set the significance level at *P* < 0.05. Soil microbial community composition (PLFAs) differences across the four treatments were evaluated using a PCA by correlation matrix (Past Version 3.25, Hammer; University of Oslo, Oslo, Norway). During the soil quality evaluation, SPSS 22.0 used both Pearson’s correlation and PCA to determine the MDS indicators and their weights. Figures were created using OriginPro 9.2 (OriginLab, Massachusetts, USA).

## Results

### Crop yield and economic benefits

**Figure 2:**
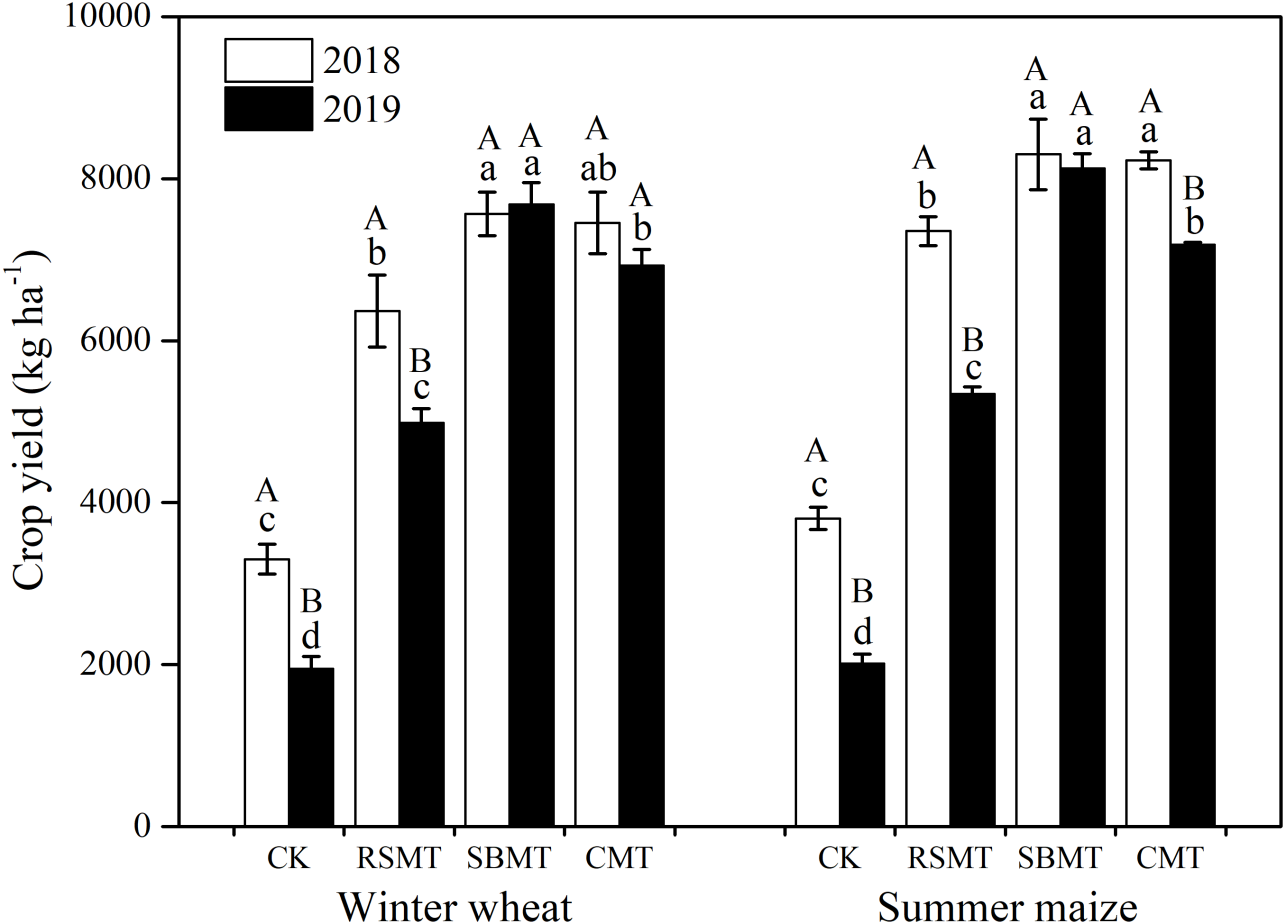
Winter wheat and summer maize yields under different fertilizer treatments in 2018 and 2019. CK, RSMT, SBMT and CMT represent no organic fertilizer treatment, rapeseed meal treatment, soybean meal treatment and cattle manure treatment, respectively. Data were means ± standard error (*n* = 3). Different lowercase letters indicate significant differences at *P* < 0.05 level among the four treatments in the same year. Different capital letters indicate significant difference at *P* < 0.05 level between 2018 and 2019 in the same treatment.

Compared to the CK group, RSMT, SBMT, and CMT showed significantly increased yields over the two-year study (*P* < 0.05, Fig. 2). In 2018, winter wheat and summer maize crop yields were 3,301.5 and 3,805.4 kg ha^−1^ in the CK group, 6,368.0 and 7,351.8 kg ha^−1^ in RSMT, 7,565.1 and 8,299.9 kg ha^−1^in SBMT, and 7,453.8 and 8,227.5 kg ha^−1^ in CMT, respectively. In 2019, winter wheat and summer maize in RSMT, SBMT, and CMT had 161%, 299%, and 256% greater yields, respectively, than the CK group (*P* < 0.05). However, the 2019 winter wheat and summer maize CK and RSMT crop yields were significantly lower than those in 2018 (*P* < 0.05).

As shown in Table 2, RSMT, SBMT, and CMT had much greater outputs than the CK group. We noted the highest annual average net income from SBMT and the lowest annual production cost from CMT. The inputs (materials, equipment, and labor) totaled $1,695.1, $2,431.0, $2,312.2 and $1,900.0/ha^−1^ for the CK, RSMT, SBMT, and CMT groups, respectively. The annual average production costs for the CK, RSMT, SBMT, and CMT groups were $0.33, $0.21, $0.15 and $0.13/kg^−1^, respectively. Excluding fertilizer and fertilization costs, the other inputs were consistent across the four treatments. RSM and SBM were priced higher than CM. However, CM required more manual labor during the fertilization process. The annual average net incomes in RSMT, SBMT, and CMT were $6,367.2, $9,318.7, and $9,047.0/ha^−1^, and were 2.70, 3.95, and 3.84 times higher than the CK group ($2,357.9/ ha^−1^), respectively. The annual average net incomes in SBMT and CMT were 1.46 and 1.42 times higher, respectively, than RSMT.

**Table 2:** Comprehensive economic benefits under different fertilizer treatments.

□	□	Details	**CK ($ ha**^−1^**)**	**RSMT ($ ha**^−1^**)**	**SBMT ($ ha**^−1^**)**	**CMT ($ ha**^−1^**)**
Input	Materials	Fertilizer	0	715.1	596.3	90.9
		Seed	194.3	194.3	194.3	194.3
		Water	222.0	222.0	222.0	222.0
	Equipment	Ploughing	177.6	177.6	177.6	177.6
		Seeding	133.2	133.2	133.2	133.2
		Harvesting	377.4	377.4	377.4	377.4
	Labor	Fertilization	0	20.8	20.8	114.0
		Irrigation	124.4	124.4	124.4	124.4
		Weed control	466.2	466.2	466.2	466.2
Annual average input			1,695.1	2,431.0	2,312.2	1,900.0
Output		2018	5,182.8	10,005.2	11,629.9	11,488.6
		2019	2,923.2	7,591.1	11,630.7	10,405.4
Annual average output			4,053.0	8,798.1	11,630.3	10,947.0
Annual average net income			2,357.9	6,367.2	9,318.1	9,047.0
Production cost ($ kg^−1^)		2018	0.24	0.18	0.15	0.12
		2019	0.43	0.24	0.15	0.13
Annual average production cost ($ kg^−1^)			0.33	0.21	0.15	0.13

**Notes.**

Data were upscaled to 1 ha based on the data collected from this experiment. US$1.0=6.756 Chinese Yuan. The outputs of wheat and maize were calculated at organic prices, respectively, $0.89/kg^−1^ and $0.59/kg^−1^. CK, RSMT, SBMT and CMT represent no organic fertilizer treatment, rapeseed meal treatment, soybean meal treatment and cattle manure treatment, respectively.

### Soil chemical characteristics

In this study, we measured 8 soil chemical characteristics (Table 3). CMT had the highest soil pH, followed by the SBMT, RSMT, and CK groups (*P* < 0.05). SBMT had the highest amounts of SOM, TN, AN, AP, and AK, followed by the CMT, RSMT, and CK groups (*P* < 0.05, Table 3). RSMT, SBMT, and CMT in 2019 had SOM and TN levels that were 54% and 71%, 102% and 90%, and 88% and 86% higher, respectively, than the CK group (*P* < 0.05). SBMT displayed the highest TP and TK levels, followed by RSMT and CMT. Soil chemical levels in 2019 were higher than those in 2018 across the three organic fertilizer treatments.

**Table 3:** Soil physicochemical and biological properties under different fertilizer treatments in 2018 and 2019.

□	□	**CK**	**RSMT**	**SBMT**	**CMT**
pH	2018	6.81 ± 0.03bA	6.92 ± 0.06bA	6.94 ± 0.03bA	7.18 ± 0.05aA
	2019	6.80 ± 0.03cA	7.01 ± 0.01bA	7.01 ± 0.01bA	7.10 ± 0.00aA
SOM (g kg^−1^)	2018	13.38 ± 0.36cA	14.89 ± 0.17bB	17.36 ± 0.66aB	16.82 ± 0.09aB
	2019	11.12 ± 0.51dB	17.16 ± 0.52cA	22.49 ± 0.67aA	20.85 ± 0.12bA
TN (g kg^−1^)	2018	0.78 ± 0.02cA	0.93 ± 0.02bB	1.07 ± 0.01aB	0.96 ± 0.02bB
	2019	0.63 ± 0.01cB	1.08 ± 0.01bA	1.20 ± 0.03aA	1.17 ± 0.01aA
TP (g kg^−1^)	2018	0.74 ± 0.03bA	0.83 ± 0.05bB	1.02 ± 0.09aA	0.81 ± 0.03bA
	2019	0.68 ± 0.03cA	0.99 ± 0.01bA	1.13 ± 0.02aA	0.98 ± 0.02bA
TK (g kg^−1^)	2018	17.25 ± 0.12bA	17.49 ± 0.15bA	18.62 ± 0.29aA	17.43 ± 0.27bB
	2019	17.04 ± 0.34bA	17.89 ± 0.15aA	18.34 ± 0.25aB	17.75 ± 0.13abA
AN (mg kg^−1^)	2018	48.15 ± 2.28cA	63.75 ± 2.73bB	79.54 ± 3.48aB	71.92 ± 4.51abB
	2019	43.04 ± 2.10dA	74.90 ± 2.56cA	90.06 ± 1.14aA	82.06 ± 2.01bA
AP (mg kg^−1^)	2018	49.22 ± 2.87dA	68.22 ± 2.87cB	109.29 ± 3.33aB	80.73 ± 3.33bB
	2019	39.53 ± 2.66dA	90.64 ± 1.18cA	122.60 ± 2.00aA	99.56 ± 1.35bA
AK (mg kg^−1^)	2018	128.76 ± 1.59dA	142.72 ± 1.64cB	162.31 ± 0.34aB	158.23 ± 0.72bB
	2019	123.28 ± 1.93cA	237.95 ± 5.00bA	263.01 ± 5.66aA	229.88 ± 0.91bA
MBC (mg kg^−1^)	2018	88.22 ± 2.11cA	115.98 ± 5.13bB	125.24 ± 0.82bB	138.76 ± 4.95aB
	2019	79.62 ± 2.98cA	137.56 ± 1.28bA	238.05 ± 3.19aA	245.54 ± 6.85aA
MBN (mg kg^−1^)	2018	12.93 ± 0.04cA	18.87 ± 1.28bB	21.79 ± 1.12abB	22.93 ± 1.26aB
	2019	13.40 ± 0.62dA	22.91 ± 0.54cA	38.10 ± 0.22bA	40.00 ± 0.66aA
Bacteria (nmol g^−1^)	2018	8.27 ± 1.39bA	9.46 ± 0.20abA	12.79 ± 1.25aA	11.42 ± 0.79abA
	2019	6.94 ± 0.94bA	9.38 ± 0.23abA	11.95 ± 1.16aA	10.70 ± 0.26aA
Fungi (nmol g^−1^)	2018	0.90 ± 0.16aA	1.01 ± 0.05aA	1.13 ± 0.11aA	1.09 ± 0.08aA
	2019	0.66 ± 0.11cA	0.94 ± 0.04bA	1.20 ± 0.08aA	1.14 ± 0.05abA
Actinomycetes (nmol g^−1^)	2018	0.55 ± 0.10bA	0.69 ± 0.19bB	1.60 ± 0.14aA	0.99 ± 0.25bA
	2019	1.00 ± 0.13bA	1.34 ± 0.05abA	1.75 ± 0.21aA	1.58 ± 0.06aA
AMF (nmol g^−1^)	2018	0.35 ± 0.07bA	0.42 ± 0.00abA	0.57 ± 0.06aA	0.54 ± 0.04aA
	2019	0.28 ± 0.05cA	0.42 ± 0.03bA	0.58 ± 0.05aA	0.52 ± 0.01abA
Total PLFA (nmol g^−1^)	2018	9.72 ± 1.52bA	11.16 ± 0.09bA	15.52 ± 1.50aA	13.50 ± 0.91abA
	2019	8.60 ± 1.18bA	11.66 ± 0.31abA	14.89 ± 1.45aA	13.42 ± 0.33aA
Soil aggregate fractions	M	41.13% ± 0.04b	57.46% ± 0.00a	60.93% ± 0.01a	59.02% ± 0.0a
	m	39.99% ± 0.02a	30.74% ± 0.01b	29.01% ± 0.03b	28.42% ± 0.01b
	S + C	18.88% ± 0.02a	11.80% ± 0.01b	10.06% ± 0.02b	12.56% ± 0.02b

**Notes.**

Data were means ± standard error (*n* = 3). Different lowercase letters indicate significant differences at *P* < 0.05 level among the four treatments in the same year. Different capital letters indicate significant difference at *P* < 0.05 level between 2018 and 2019 in the same treatment. CK, RSMT, SBMT and CMT represent no organic fertilizer treatment, rapeseed meal treatment, soybean meal treatment and cattle manure treatment, respectively.

SOM soil organic matter TN total nitrogen TP total phosphorus TK total potassium AN available nitrogen AP available phosphorus AK available potassium MBC microbial biomass carbon MBN microbial biomass nitrogen AMF arbuscular mycorrhizae fungi M large macroaggregate + small macroaggregate m microaggregate S + C silt + clay

### Soil aggregate fractions

Large and small macroaggregates were the most represented aggregate-size classes. We noted that SBMT had the highest amount of macroaggregates, while the CK group had the highest amounts of microaggregates and silt + clay (*P* < 0.05). The proportion of macroaggregates in RSMT, SBMT, and CMT were 40%, 48%, and 43% greater, respectively, than in the CK group. The proportion of microaggregates in RSMT, SBMT, and CMT were 23%, 27%, and 29% lower, respectively, and the proportion of silt + clay was 38%, 47%, and 33% lower, respectively, than in the CK group (*P* < 0.05, Table 3).

### Soil biological characteristics

Organic fertilizers significantly increased soil MBC and MBN levels (*P* < 0.05, Table 3). In 2018 and 2019, CMT had the highest MBC and MBN levels, followed by SBMT and RSMT. We found the lowest MBC and MBN levels in the CK group. At the end of the experiment, MBC levels in RSMT, SBMT, and CMT were 73%, 199%, and 208% (*P* < 0.05) greater, respectively, than in the CK group. MBN levels in RSMT, SBMT, and CMT were 71%, 184%, and 199% greater, respectively, than in the CK group (*P* < 0.05). RSMT, SBMT, and CMT had MBC levels in 2019 that were 19%, 90%, and 77% greater, respectively, than MBC levels in 2018, and MBN levels in 2019 that were 21%, 75%, and 74% greater, respectively, than MBN levels in 2018 (*P* < 0.05, Table 3).

The total amount of PLFAs, bacteria, fungi, actinomycetes, and AMF in 2018 and 2019 were highest in SBMT, followed by the CMT, RSMT, and CK groups (Table 3). At the end of the experiment, RSMT, SBMT, and CMT had 36%, 73%, and 56% more PLFAs; 35%, 72%, and 54% more bacteria; 43%, 83%, and 74% more fungi; 34%, 74%, and 58% more actinomycetes; and 52%, 106%, and 86% more AMF, respectively, than the CK group (*P* < 0.05, Table 3).

**Figure 3:**
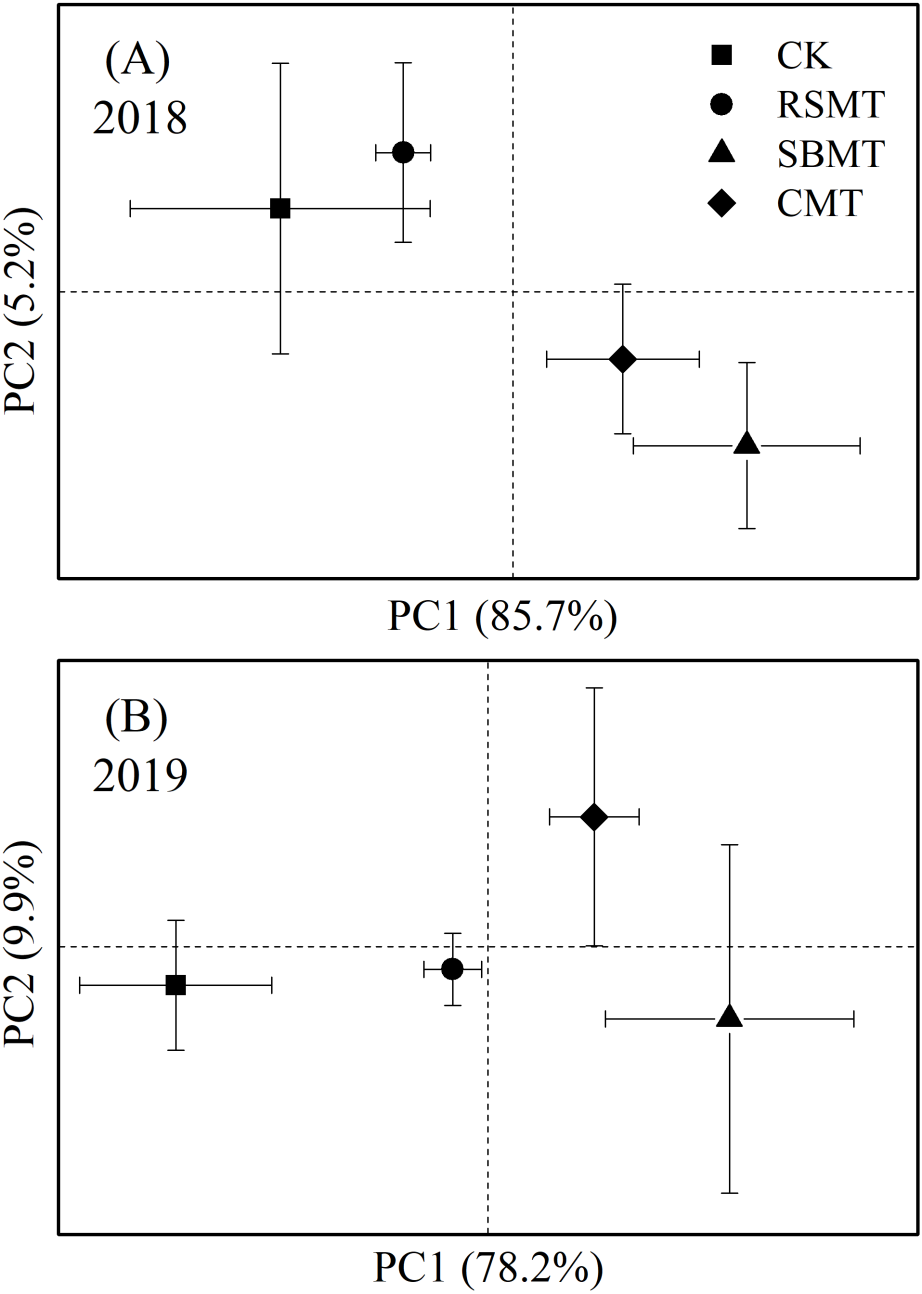
PCA for PLFAs in topsoil samples under different fertilizer treatments in 2018 (A) and 2019 (B). In 2018, PC1 and PC2 accounted for 85.7% and 5.2% of the total variance, respectively; In 2019, PC1 and PC2 accounted for 78.2% and 9.9% of the total variance, respectively. CK, RSMT, SBMT and CMT represent no organic fertilizer treatment, rapeseed meal treatment, soybean meal treatment and cattle manure treatment, respectively. Data were means ± standard error (*n* = 3).

We performed 27 PLFAs to PCA in 2018 and in 2019 (Fig. 3). In 2018, principle component 1 (PC1) and principle component 2 (PC2) accounted for 85.7% and 5.2% of the total variance, respectively. SBMT and CMT demonstrated a clear separation along PC1 and PC2 from the RSMT and CK groups (Fig. 3A). In 2019, the first two PCs accounted for 78.2% and 9.9% of the total variance, respectively. Microbial communities from the CK group and the three organic fertilizer treatments showed significant separation in PC1 (*P* < 0.05). In PC2, CMT was isolated from the other three treatments (*P* > 0.05, Fig. 3B).

### Soil quality evaluation

The PCA results (Table 4) showed that only the first two PCs had eigenvalues >1.0 and 90.917% of the total variability: 82.253% for PC1 and 8.664% for PC2. In PC1, the highly loaded indicators were SOM, TN, TP, AN, AP, AK, MBC, MBN, bacteria, fungi, AMF, total PLFA, and macroaggregates (Table 4). These 13 indicators showed significant correlations among each other (*r* > 0.70). Since the SOM had the highest absolute loading sum, it was selected for the MDS (Table 5). The highly loaded indicators in PC2 were TK, bacteria, actinomycetes, and total PLFA. We also selected actinomycetes for the SQI MDS because they had the highest absolute correlation coefficient (Table 5).

**Table 4:** Loading, eigenvalue and variance explained of principal component analysis (PCA) using eighteen soil properties.

Soil quality indicators	PC1	PC2
pH	0.859	−0.119
SOM	** 0.977**	−0.018
TN	**0.968**	−0.132
TP	**0.953**	−0.106
TK	0.778	**−0.415**
AN	**0.979**	−0.159
AP	**0.977**	−0.066
AK	**0.950**	−0.175
MBC	**0.923**	0.059
MBN	**0.923**	0.061
Bacteria	**0.883**	**0.445**
Fungi	**0.906**	0.398
Actinomycetes	0.862	**0.457**
AMF	**0.920**	0.352
Total PLFA	**0.886**	**0.444**
M	**0.907**	−0.386
Microaggregate	−0.848	0.392
Silt + clay	−0.793	0.296
Eigenvalue	14.806	1.559
Explained variance (%)	82.253	8.664
Cumulative variance (%)	82.253	90.917

**Notes.**

Boldface factor loadings are considered highly weighted. Boldface and underlined loadings correspond to the indicators retained in the MDS.

SOM soil organic matter TN total nitrogen TP total phosphorus TK total potassium AN available nitrogen AP available phosphorus AK available potassium MBC microbial biomass carbon MBN microbial biomass nitrogen AMF arbuscular mycorrhizae fungi M large macroaggregate + small macroaggregate

**Table 5:** Person correlation coefficients for highly weighed variables under PC1 and PC2 (*n* = 12).

□	**SOM**	**TN**	**TP**	**TK**	**AN**	**AP**	**AK**	**MBC**	**MBN**	**B**	**F**	**A**	**AMF**	**TPLFA**	**M**
**SOM**	1	0.952^**^	0.926^**^	0.724^**^	0.958^**^	0.970^**^	0.904^**^	0.945^**^	0.950^**^	0.841^**^	0.867^**^	0.808^**^	0.877^**^	0.843^**^	0.886^**^
**TN**		1	0.936^**^	0.723^**^	0.979^**^	0.965^**^	0.967^**^	0.884^**^	0.889^**^	0.781^**^	0.835^**^	0.740^**^	0.828^**^	0.784^**^	0.913^**^
**TP**			1	0.822^**^	0.955^**^	0.981^**^	0.965^**^	0.810^**^	0.808^**^	0.817^**^	0.811^**^	0.782^**^	0.829^**^	0.815^**^	0.884^**^
**TK**				1	0.818^**^	0.787^**^	0.809^**^	0.639^**^	0.625^**^	0.540^**^	0.536^**^	0.566^**^	0.597^**^	0.545^**^	0.878^**^
**AN**					1	0.981^**^	0.966^**^	0.898^**^	0.896^**^	0.792^**^	0.821^**^	0.759^**^	0.841^**^	0.793^**^	0.937^**^
**AP**						1	0.963^**^	0.881^**^	0.879^**^	0.838^**^	0.852^**^	0.797^**^	0.865^**^	0.837^**^	0.888^**^
**AK**							1	0.799^**^	0.796^**^	0.771^**^	0.804^**^	0.737^**^	0.816^**^	0.772^**^	0.914^**^
**MBC**								1	0.998^**^	0.796^**^	0.847^**^	0.782^**^	0.861^**^	0.803^**^	0.804^**^
**MBN**									1	0.797^**^	0.850^**^	0.783^**^	0.854^**^	0.804^**^	0.806^**^
**B**										1	0.970^**^	0.987^**^	0.972^**^	0.999^**^	0.639^**^
**F**											1	0.963^**^	0.979^**^	0.975^**^	0.668^**^
**A**												1	0.960^**^	0.990^**^	0.625^**^
**AMF**													1	0.975^**^	0.710^**^
**TPLFA**														1	0.642^**^
**M**															1

**Notes.**

** indicates *P* < 0.01.

SOM soil organic matter TN total nitrogen TP total phosphorus TK total potassium AN available nitrogen AP available phosphorus AK available potassium MBC microbial biomass carbon MBN microbial biomass nitrogen B bacteria F fungi A actinomycetes AMF arbuscular mycorrhizae fungi TPLFA total PLFA M large macroaggregate + small macroaggregate

**Figure 4:**
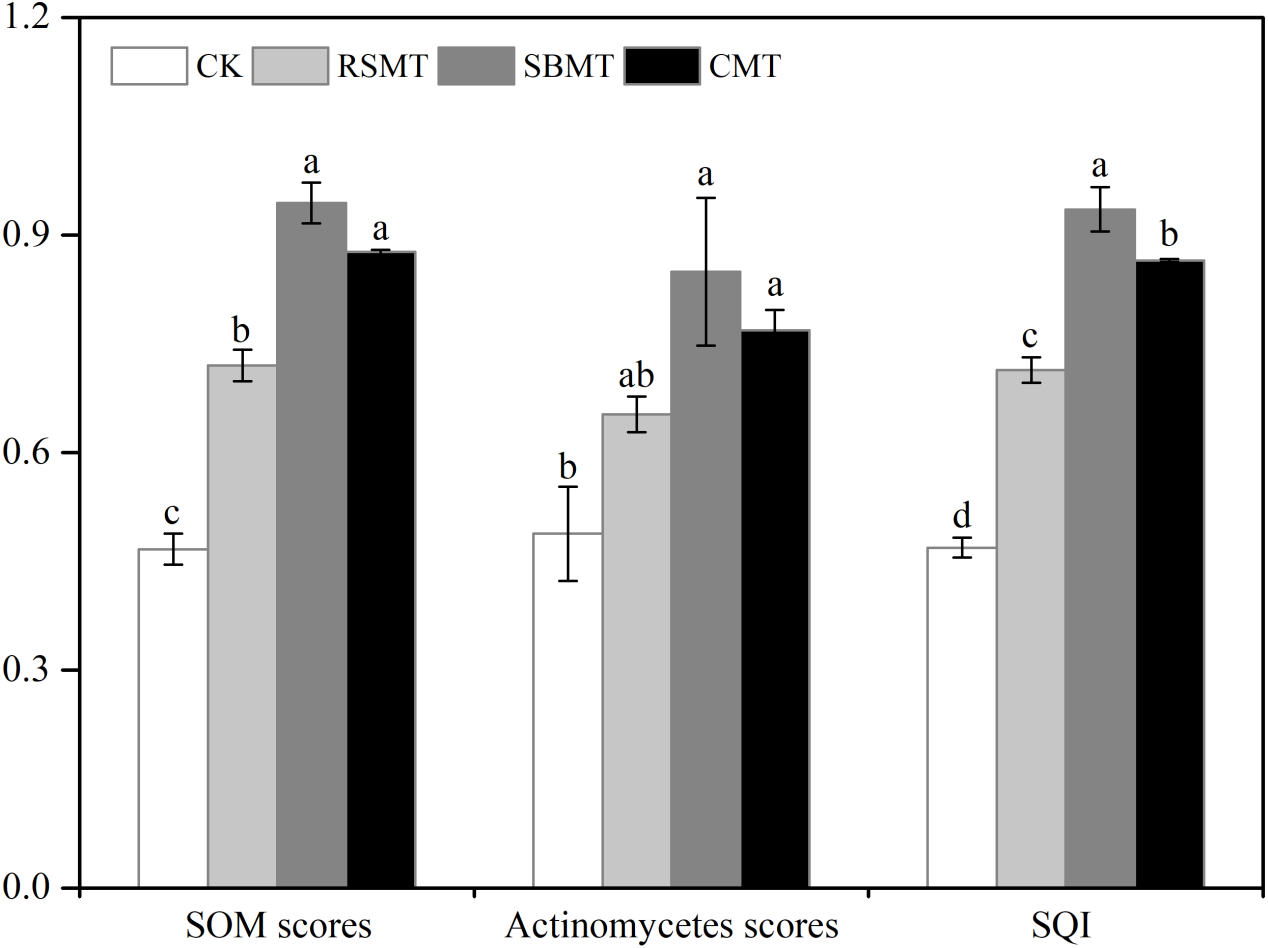
Scored values of MDS indicators (SOM, actinomycetes) and soil quality index. CK, RSMT, SBMT and CMT represent no organic fertilizer treatment, rapeseed meal treatment, soybean meal treatment and cattle manure treatment, respectively. Data were means ± standard error (*n* = 3). Different lowercase letters indicate significant differences at *P* < 0.05 level among the four treatments. SOM, soil organic matter; SQI, soil quality index.

After selecting SOM and actinomycetes for the MDS, we applied a “more is better” linear scoring function. The highest SOM and actinomycete score values were found in SBMT, followed by CMT, RSMT, and CK (Fig. 4). We determined the weight of each MDS indicator by calculating its contribution percentage in each PC. The weights of SOM and actinomycetes were 0.905 and 0.095, respectively. After scoring the selected MDS indicators, we calculated the SQI using the following weighted equation:

(3) \begin{eqnarray*}SQI=0.905\times {S}_{SOM}+0.095\times {S}_{Actinomycetes}\end{eqnarray*}

The average SQI values for the CK, RSMT, SBMT, and CMT groups were 0.47, 0.71, 0.94, and 0.87, respectively. The differences across the four treatments were significant (*P* < 0.05, Fig. 4).

## Discussion

### Crop yield and economic benefits

Previous studies have claimed that using organic fertilizers results in an average of 19.8–25% lower crop yield than chemical fertilizers (Ponti, Rijk & Ittersum, 2012; Seufert, Ramankutty & Foley, 2012; Ponisio et al., 2015). However, yield losses have not been the case across all climate zones, organic fertilizers, or crops (Seufert, Ramankutty & Foley, 2012; Gattinger et al., 2013). When using best management practices and suitable organic fertilizers, an organic system’s yield can meet or even surpass a chemical system (Liu et al., 2016). Our results clearly indicate that wheat and maize in SBMT and CMT produced much higher yields than the average in Shandong Province (6,035.0 kg ha^−1^ for wheat and 6,469.0 kg ha^−1^ for maize). Therefore, we suggest that the proper application of SBM and CM in a winter wheat-summer maize rotation system would reduce yield losses.

For many farmers, however, crop yield benefits are second to economic benefits. In our previous studies, we found that organic systems presented much higher economic benefits than conventional systems (Liu et al., 2016; Meng et al., 2016). Using economic benefit analysis, we observed the highest input in RSMT, and the lowest annual production costs in CMT, followed by SBMT. Net incomes were higher for SBMT ($9,318.1/ha^−1^) and CMT ($9,047.0/ha^−1^), and they were 1.46 and 1.42 times greater, respectively, than RSMT, as well as 4.82 and 4.68 times greater, respectively, than conventionally farmed local crops (Liu et al., 2016). Our results indicate that SBM and CM fertilizers generate more economic benefits than RSM and chemical fertilizers.

### Soil chemical characteristics

We found that soil pH in RSMT, SBMT, and CMT was significantly higher than in the CK group (*P* < 0.05), indicating that organic fertilizers, specifically CM, can alleviate the soil acidification caused by long-term chemical fertilizer application (Guo et al., 2010; Zhu et al., 2018). At the end of our study, we found much higher concentrations of SOM and the minerals essential for plant growth (N, P, and K) in organic fertilizer treatments than in the CK group, which corresponded with the results of Liu et al. (2016). The soil chemical levels for the three organic fertilizer treatments in 2019 were significantly higher than those in 2018, indicating organic fertilizers’ ability to nurse cultivated land. This effect was more obvious over time.

### Soil aggregate fractions

Soil aggregates are important indicators of soil quality and the effects of soil management (Barthès & Roose, 2002). Organic fertilizer treatments showed significantly higher proportions of macroaggregates and lower levels of microaggregates and silt + clay than the CK group, which was consistent with the findings of Liao, Boutton & Jastrow (2006) and Yu et al. (2012). These results may be due to the higher amounts of AMF and other fungi (Li et al., 2020) that predominantly proliferate in larger soil pores and contribute to macroaggregate formation (De Gryze et al., 2005; Wilson et al., 2009; Ding & Han, 2014).

### Soil biological characteristics

MBC representing about 1–5% of the soil’s TOC could provide an effective warning of the soil quality (Powlson & Brooks, 1987). Our results clearly demonstrated that organic fertilizers significantly increased MBC and MBN levels, which was consistent with the findings of Kaur, Kapoor & Gupta (2005), who found that MBC levels tended to be lower in unfertilized soil compared to soil treated with organic fertilizers. Increased MBC and MBN levels may be caused by organic fertilizers’ abundant metabolizable carbon and N that promote root growth and increase soil microbial biomass (Ekblad & Nordgren, 2002). More importantly, organic fertilizers directly supply energy to microbes, further increasing soil microbial biomass and activity (Vance & Chapin, 2001; Ekblad & Nordgren, 2002).

Our PCA results indicated that organic fertilizers significantly affected the soil microbial community. SBMT and CMT showed higher amounts of total PLFAs, bacteria, fungi, and actinomycetes than RSMT and the CK group. Bacteria represented about 80–85% of the total PLFAs in this study. The increase of bacteria in SBMT and CMT had a positive effect on soil properties, owing to the fact that many types of bacteria (G+ and G–) can easily adapt to environmental changes (McCann et al., 1994; Zhang et al., 2018). G+ and G–produce spores, have thicker cell walls, and can adjust their cell wall structure, making them more adept at surviving environmental changes (McCann et al., 1994; Zhang et al., 2018). Therefore, SBM and CM significantly affected soil properties by changing the soil’s microbial communities.

AMF are an important group of soil microorganisms. In this study, SBMT and CMT had more AMF than RSMT or the CK group, which corresponded with previous research (Oehl et al., 2004; Manoharan et al., 2017). AMF help protect plants against soil pathogens (Sikes, Cottenie & Klironomos, 2009), increase plant drought tolerance (Auge et al., 2001), and provide plants with nutrients (Van der Heijden et al., 1998; Hill et al., 2010; Wagg et al., 2011), meaning that the lower amounts of AMF found in RSMT and CK groups may lead to reduced agricultural land quality and nutrient utilization in the follow-up crops.

### Soil quality

In this study, we selected 18 indicators as potential soil quality indicators. Previous studies have also adopted an MDS when using an SQI (Qiu et al., 2019; Boafo et al., 2020), with the most commonly selected indicators being SOM, TN, and MBC (Abid & Lal, 2008; Liu et al., 2014; Bera et al., 2016). Selecting MDS indicators is contingent on various factors. In this study, we selected SOM and actinomycetes as MDS indicators. The SQI values varied across the four treatments and ranged from 0.47 to 0.94. The highest SQI value occurred in SBMT, followed by CMT and RSMT, indicating that SBM application is the most capable at maintaining soil quality.

## Conclusions

Using a constant total N application rate, we compared RSMT, SBMT, and CMT to determine which fertilizer achieved the highest crop yields, economic growth, and levels of SOM and essential minerals (N, P, and K). We found the highest MBC and MBN levels in CMT, and the highest amount of PLFAs in SBMT. We selected SOM and actinomycetes as the MDS properties, and observed the highest SQI values in SBMT, followed by CMT and RSMT. Our results demonstrated that SBM and CM organic fertilizers may be better than RSM at improving crop yields and soil quality. This study provides a scientific basis for selecting effective organic fertilizers that can replace chemical fertilizers.

##  Supplemental Information

10.7717/peerj.9668/supp-1 Supplemental Information 1 The raw data of figures and tables

## Additional Information and Declarations

### Field Study Permissions

The following information was supplied relating to field study approvals (i.e., approving body and any reference numbers): Field experiment was approved by the Plant Eco-physiological Research Group (PERG) in the Institute of Botany, Chinese Academy of Sciences (project number: 201707.8). In this study, the name of the farmer is Jianjin Jiang who has a verbal agreement with the PERG in the Institute of Botany, Chinese Academy of Sciences. Although the farmland belongs to Jianjin Jiang, he has already rented it to the PERG. So, we should only get permission of the PERG. Any other permission from the individual and company is not needed.

### Data Availability

The following information was supplied regarding data availability: The raw measurements are available as a Supplemental File.
